# Leukocyte telomere length in patients with myotonic dystrophy type I: a pilot study

**DOI:** 10.1002/acn3.50954

**Published:** 2019-12-05

**Authors:** Youjin Wang, Ana Best, Roberto Fernández‐Torrón, Rotana Alsaggaf, Mikel Garcia‐Puga, Casey L. Dagnall, Belynda Hicks, Mone’t Thompson, Ander Matheu Fernandez, Miren Zulaica Ijurco, Mark H. Greene, Adolfo Lopez de Munain, Shahinaz M. Gadalla

**Affiliations:** ^1^ Clinical Genetics Branch Division of Cancer Epidemiology and Genetics National Cancer Institute National Institutes of Health Bethesda Maryland; ^2^ Biostatistics Branch Division of Cancer Epidemiology and Genetics National Cancer Institute National Institutes of Health Bethesda Maryland; ^3^ Centro de Investigación Biomédica en Red sobre Enfermedades Neurodegenerativas Institute Carlos III Madrid Spain; ^4^ Neuroscience Area Institute Biodonostia San Sebastian Spain; ^5^ Cancer Genomics Research Laboratory Division of Cancer Epidemiology and Genetics National Cancer Institute National Institutes of Health Bethesda Maryland; ^6^ Cancer Genomics Research Laboratory Leidos Biomedical Research, Inc. Frederick National Laboratory for Cancer Research Frederick Maryland; ^7^ Oncology Area Biodonostia Health Research Institute San Sebastián Spain; ^8^ Department of Neurology Hospital Universitario Donostia San Sebastian Spain; ^9^ Department of Neurosciences Universidad del País Vasco UPV‐EHU San Sebastian Spain

## Abstract

Myotonic dystrophy type I (DM1) is an autosomal dominant disease of which clinical manifestations resemble premature aging. We evaluated the contribution of telomere length in pathogenesis in 361 DM1 patients (12 with serial measurements) and 223 unaffected relative controls using qPCR assay. While no differences in baseline leukocyte relative telomere length (RTL) was noted, the data suggested an accelerated RTL attrition in DM1 (discovery cohort: T/S change/year = −0.013 in DM1 *vs. *−0.005 in controls, *P* = 0.04); similar trend was noted in validation cohort. Further investigations are needed to examine the role of TL in the pathophysiology of DM1.

## Introduction

Myotonic dystrophy type 1 (DM1) is an autosomal dominant multisystem disorder resulting from an unstable trinucleotide (CTG)_n_ repeat expansion in the 3’ untranslated region of the *dystrophia myotonica‐protein kinase* (*DMPK*) gene.^1^ Clinical manifestations of DM1 include progressive muscle weakness and wasting, myotonia, premature cataracts, cardiac conduction defects, gastrointestinal/endocrine abnormalities, and increased risk of certain cancers.^2^, ^3^ It has been suggested that the DM1 phenotypes may resemble a premature aging syndrome.^4^

Telomeres are tandem hexanucleotide (TTAGGG)_n_ repeats and protein complexes at the end of chromosomes, maintaining genomic stability.^5^ They shorten with each cell division^6^ and are marker of cellular aging. Few studies suggested a link for telomere length in the pathogenesis of Duchenne (DMD) or limb girdle muscular dystrophies.^7^, ^8^ Telomere length studies in DM1 were limited; an *in vitro* investigation using DM1 muscle precursor cells from three fetuses with congenital DM1 showed abnormal accumulation of P16 and higher telomere attrition per cell division compared with controls.^9^

Here we compared leukocyte relative telomere length (RTL) in a large cohort of genetically‐ confirmed DM1 patients and DM1 mutation‐free clinically healthy relative controls and evaluated telomere length attrition overtime in a subset of patients with serial blood samples.

## Materials and Methods

### Study participants

We used data and samples from 379 DM1 patients and 235 DM1‐free relative controls from the Spanish Guipúzcoa historical myotonic dystrophy cohort.^10^ Participants’ demographics and clinical information were obtained through medical records review. We excluded 30 individuals (28 missing age, one twin, and one with failed quality control metrics for RTL assay). The final analysis included 361 DM1 patients and 223 controls. Twelve participants provided a second sample for RTL measurement, as convenience permitted.

The study was approved by the Donostia University Hospital Ethical Board and by the Office of Human Subjects Research Protections at the National Institutes of Health.

### DNA extraction and telomere length measurement

Blood DNA was extracted using the salting out procedure or Qiagen Flexigen DNA kit (QIAGEN, Germantown, MD). We measured RTL after adapting the originally published quantitative polymerase chain reaction (qPCR) method.^11^ Method details are available elsewhere.^12^ Briefly, qPCR assay measures a ratio between telomere repeats amplification (T) to that of an autosomal single‐copy gene (S; *36B4*). The T/S ratio then get normalized using internal QC calibrator samples to yield a standardized T/S ratio. All samples were assayed in triplicate. The coefficient of variation for internal control samples was 4.5%.

### Statistical analysis

All analyses were stratified by DNA extraction method to account for known variable qPCR sensitivity to extraction method.^13^ Samples extracted by salting out (*N* = 223 patients and 162 controls) served as a discovery cohort, and those extracted by Qiagen Flexigen DNA kit (*N* = 138 patients and 61 controls) served as a validation cohort. RTL data from serial samples extracted by the same method were available for 12 DM patients (*N* = 7 for discovery, *N* = 5 for validation cohort).

We used generalized linear models to compare the mean RTL between DM1 and controls, and in DM1 patients by CTG repeat size categories (40–50, 51–249, 250–499, ≥500). A random intercept linear mixed model was used for repeated measurement analysis; RTL annual attrition rate in DM1 patients with repeated samples (median time between samples = 9 years, range = 4‐17 years in the discovery, and median time = 3 years, range = 2–3 years in the validation) was compared with that expected from regression models of RTL and age using control cross‐sectional data using R package ‘lme4’. Differences in RTL attrition rates between DM1 patients and controls was assessed by an interaction term between DM1‐control status and age. Statistical analyses were conducted using SAS version 9.4 and R version 3.4.4.

## Results

### Characteristics of study participants

DM1 patients and controls showed similar ages (median age = 42.0 and 43.0 years in DM1 *vs.* controls, respectively) and sex distributions (proportion of males = 45.7% and 43.9% in DM1 *vs.* controls, respectively). No age or sex differences were noted between DM1 patients in the two study subsets, or between patients and relatives in each set (*P* > 0.05). Patients with DM1 in the discovery and validation cohorts also had similar CTG repeat size (median repeat size = 433 and 500 for discovery *vs.* validation cohorts, respectively, *P* = 0.37) (Table S1).

In DM1 patients, the size of CTG repeat was inversely correlated with age at genetic testing in both discovery (*r *= −0.26, *P* = 0.0003) and validation cohorts (*r *= −0.21, *P* = 0.02) (Fig. S1A‐B).

The patients with a second blood sample showed similar age, and sex distribution. However, despite of lack of statistical significance, the data suggested that patients with subsequent sample may have a less severe disease as evident by shorter CTG repeat sizes (median = 167 *vs.* 500, in those with and without subsequent sample, respectively) and less Muscular Impairment Rating Scale (MIRS) (0% *vs.* 24% with MRIS > 3, respectively) (Table S2).

### Telomere length in DM1 patients and controls

RTL was inversely correlated with age among both DM1 patients and controls (discovery cohort: *r *= −0.39, *P* < 0.0001 for DM1 and *r *= −0.51, *P* < 0.0001 for controls; validation cohort: *r *= −0.27, *P* = 0.002 for DM1 and *r *= −0.23, *P* = 0.07 for controls; Fig. S2A‐B).

**Figure 1:**
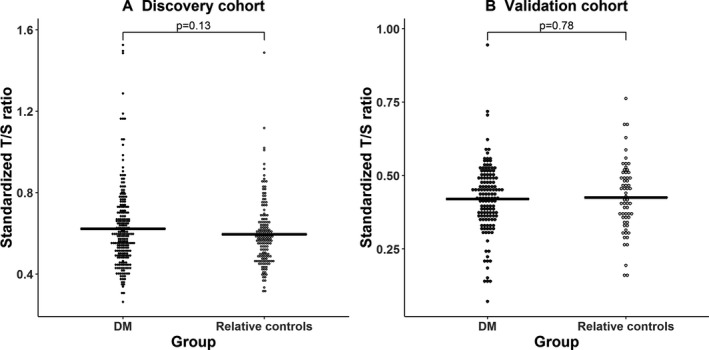
Comparison of relative telomere length between DM1 patients and their healthy relative controls. (A) Discovery cohort, (B) Validation cohort.

**Figure 2:**
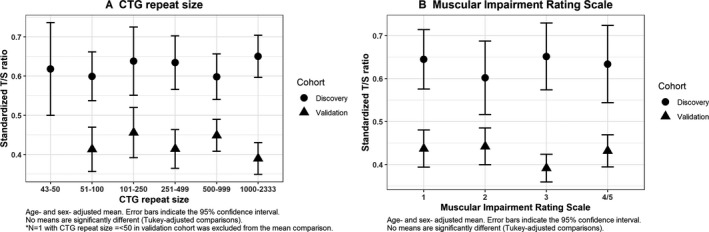
Relative telomere length in DM1 patients by CTG repeat size and by Muscular Impairment Rating Scale (MIRS) in age‐ and sex‐adjusted models. (A) CTG repeat size, (B) Muscular Impairment Rating Scale.

No differences were observed in RTL between DM1 patients and controls at baseline: discovery cohort: (mean T/S ± SD) = 0.62 ± 0.20 and 0.60 ± 0.16 for DM1 and controls, respectively, *P* = 0.13; or in the validation cohort: (mean T/S ± SD)=0.42 ± 0.12 and 0.43 ± 0.12, respectively, *P* = 0.78, Fig. 1A and B). Age‐ and sex‐adjustment did not alter the results. In DM1 patients, RTL was not associated with CTG repeat expansion size, nor with MIRS in models adjusted for age and sex (Fig. 2A and B).

### Telomere length attrition overtime in patients with DM1

Baseline RTL was similar in patients with and without repeated RTL measurements (median T/S = 0.58 *vs.* 0.52, *P* = 0.27).

**Figure 3:**
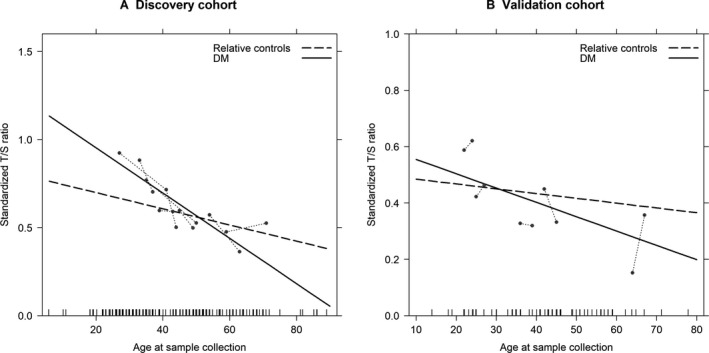
Comparison of annual relative telomere length attrition between DM1 patients and their healthy relative controls. (A) Discovery cohort, (B) Validation cohort.

In the repeated RTL measurement analyses, and when compared with expected based on RTL attrition from relatives’ cross‐sectional data, a more rapid RTL decline was noted in DM1 in the discovery cohort (T/S decline/year = −0.013 *vs. *−0.005 for DM1 and controls, respectively, *P* = 0.04). A similar (non‐significant) trend was noted in the validation cohort (T/S decline/year = −0.005 *vs. *−0.002, *P* = 0.33) (Fig. 3A and B, Table S3).

## Discussion

In this analysis of a large cohort of patients with genetically confirmed DM1 and clinically healthy relative controls, we observed no difference in leukocyte RTL measured at genetic testing between patients and controls, yet, a higher rate of annual telomere length attrition was noted in DM1 patients.

Prior DM1 telomere length studies have been limited to few small *in‐vitro* investigations focusing on its possible role in muscle precursor cell dysfunction. In agreement with our results of similar leukocyte RTL in DM1 and controls, a study comparing telomere length in distal (with clinical and pathological alterations) and proximal muscles (relatively spared) from four DM1 patients showed no telomere length difference.^14^ Yet, we showed a higher rate of RTL attrition overtime in DM1 patients than expected based on RTL attrition rate from controls. Our findings were also consistent with a study of muscle satellite cells in fetuses/infants with congenital DM1 which showed a faster telomeric DNA loss per cell division (171.9 ± 17 base pairs) when compared with control cells (108.1 ± 10 base pairs); the authors concluded that large CTG expansion repeats may interfere with telomere homeostasis in those patients.^9^ Of note, we found no association between patients’ leukocyte RTL and CTG repeat size; suggesting no direct role for repeat expansion size in the observed telomeric attrition. However, our data were limited by the unavailability of progenitor repeat size. Also, testing this hypothesis in blood cell (not known to be affected in DM1), may not be directly applicable to somatic changes in affected organs such as the muscles. Somatic mosaicism and instability in repeat expansion size are well‐described in DM1.^15^ It is possible that the accelerated telomere attrition observed in our DM1 patients is a consequence of the cellular exposure to oxidative stress. The triple‐guanine sequence in telomeric DNA (TTAGGG)_n_ is very sensitive to oxidative damage,^16^ resulting in accelerated telomere shortening.^17^ Previous studies have shown a significant increase in blood level of free radicals and oxidative stress biomarkers in DM1 patients,^18^, ^19^ suggesting a role for oxidative stress in disease progression.

The observed differences in the rate of TL attrition between the discovery and validation cohort could be affected by the difference in the time interval between samples (median interval = 9 years in the discovery, and 3 years in the validation). It has been shown that TL elongation in longitudinal studies is mostly artifact related to measurement errors in studies with short follow‐up (≤5 years) where TL changes are usually small and hard to capture.^20^

Our study strengths include a relatively large sample size, the genetic diagnosis confirmation, the objectively healthy status of controls, and the availability of repeat expansion size. Limitations include the small sample size of patients with serial samples, who were not systematically sampled. Due to short time period between blood sampling, observed TL elongation in some patients could be artifactual.^20^ Our control data were based on baseline measurement of RTL in relatives because of the unavailability of serial samples; a prior study has shown small differences in TL attrition rate calculated from cross‐sectional or longitudinal data in the general population (24.6 *vs.* 31 bp per year).^20^ The qPCR assay is sensitive to pre‐analytic processing, notably DNA extraction method. Consequently, we presented our results stratified by DNA extraction method. Although similar trends were observed in the two datasets, we cannot rule out the possibility that the difference in DNA extraction methods and/or time between serial samples contributed to the observed difference in the rate of TL attrition between the two sets.

In conclusion, our results suggest that telomere shortening does not play a direct role in DM1 etiology but may be a consequence of molecular alterations over time, possibly secondary to oxidative stress. It is possible that accelerated telomere shortening may mediate the age‐related phenotypic presentation of DM1.

## Conflict of Interest

The authors declare no conflict of interest.

## Supporting information


**Figure S1**. Correlation between CTG repeat size and age in DM1 patients. A. Discovery cohort, B. Validation cohort
**Figure S2**. Correlation between relative telomere length and age. A. Discovery cohort, B. Validation cohort
**Table S1**. Baseline characteristics of DM1 patients and their unaffected relative controls, stratified by DNA extraction method
**Table S2**. Comparison of baseline characteristics of DM1 patients with and without repeated relative telomere length measurement
**Table S3**. Telomere length attrition in DM1 patients and controls
