# Supplementary material for: Leukocyte telomere length in patients with myotonic dystrophy type I: a pilot study
Source: Ann Clin Transl Neurol. 2019 Dec 5;7(1):126–31. doi: 10.1002/acn3.50954 (PMC6952307; doi:10.1002/acn3.50954)
Supplement: Supplementary file 1 — Figure S1. Correlation between CTG repeat size and age in DM1 patients. A. Discovery cohort, B. Validation cohort Figure S2. Correlation between relative telomere length and age. A. Discovery cohort, B. Validation cohort Table S1. Baseline characteristics of DM1 patients and their unaffected relative controls, stratified by DNA extraction method Table S2. Comparison of baseline characteristics of DM1 patients with and without repeated relative telomere length measurement Table S3. Telomere length attrition in DM1 patients and controls [file ACN3-7-126-s001.docx]

**SUPPLEMENTARY MATERIALS**

**Supplemental Figure 1. Correlation between CTG repeat size and age in DM1 patients**

| **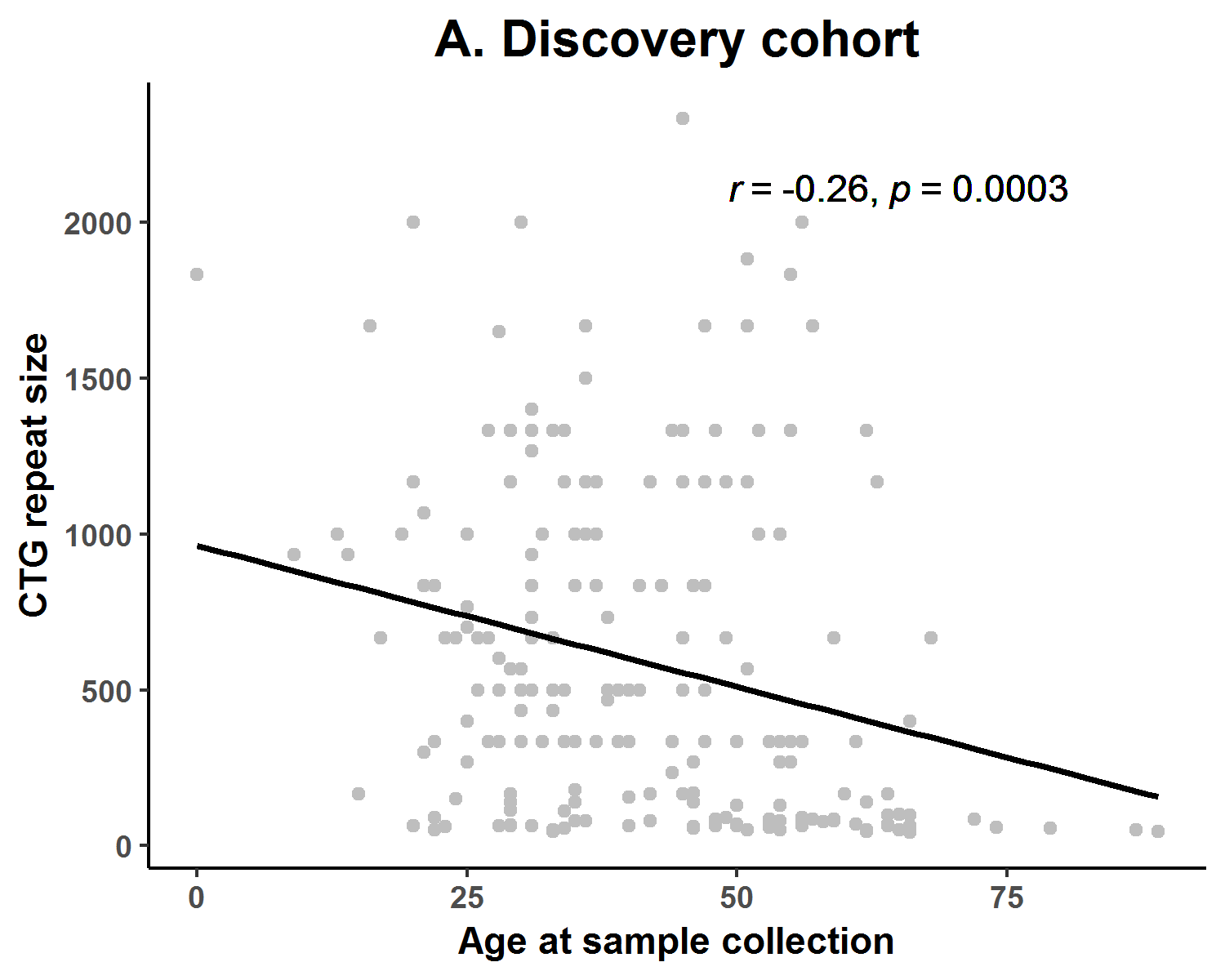** | **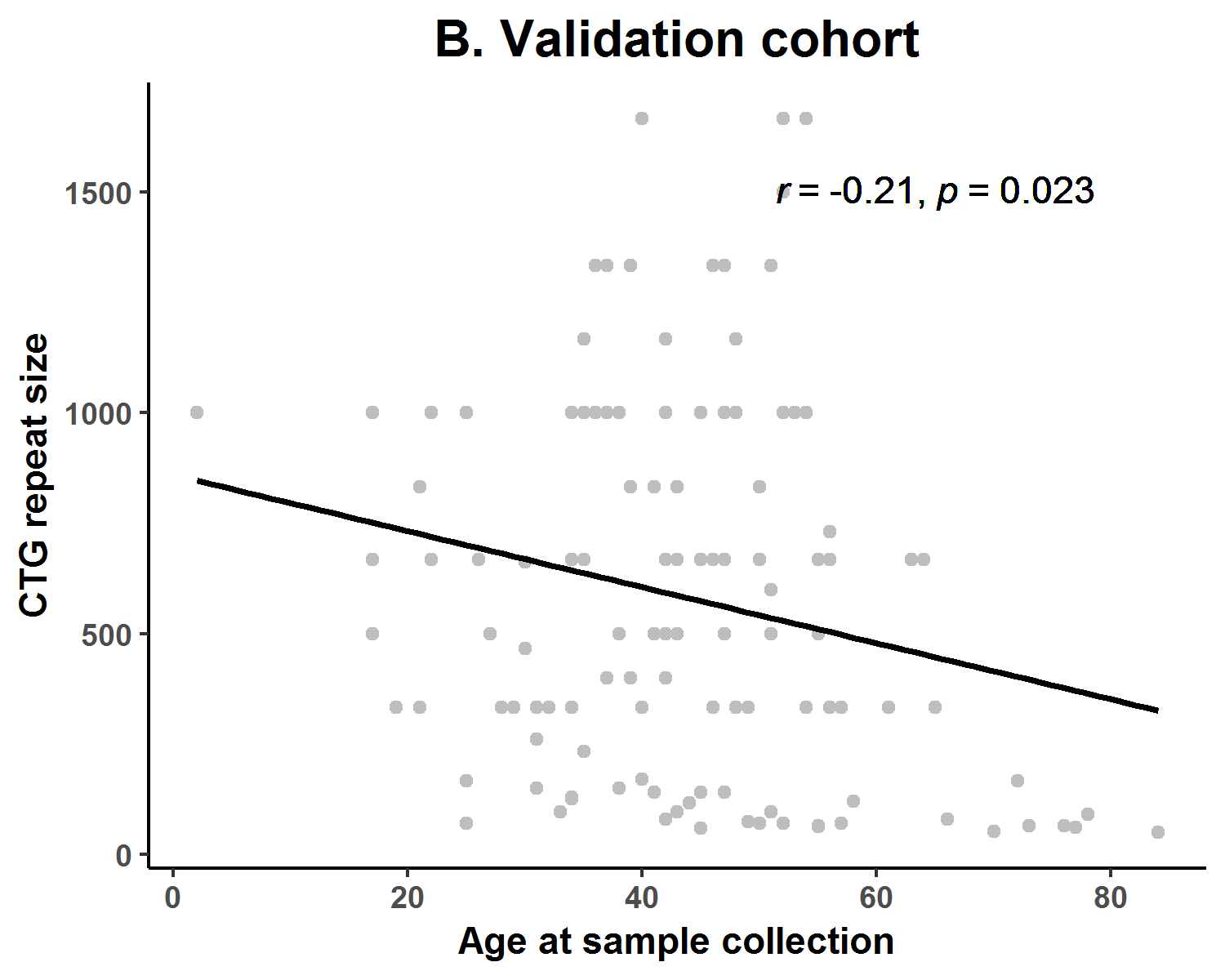** |
| --- | --- |

**Supplemental Figure 2. Correlation between relative telomere length and age**

| **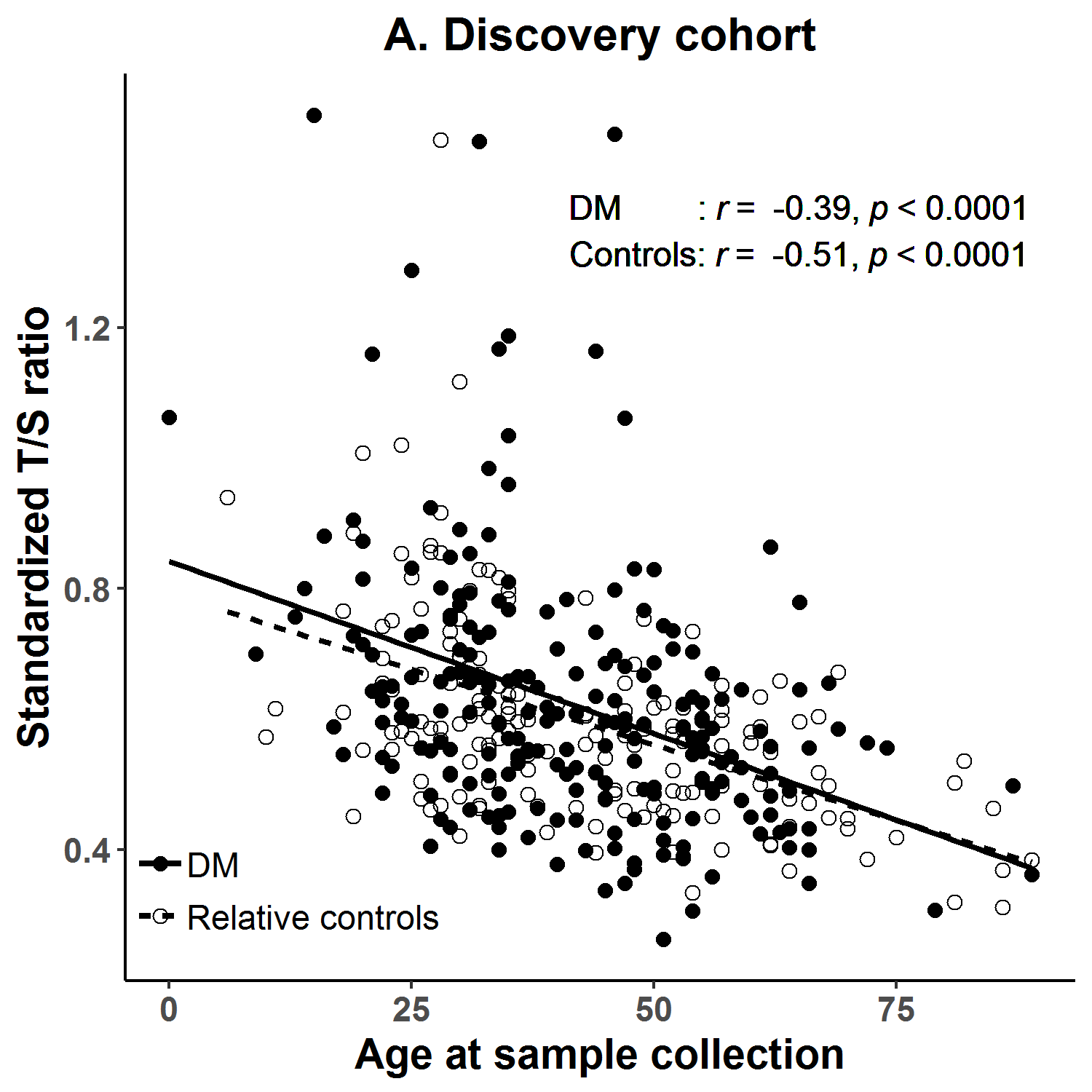** | **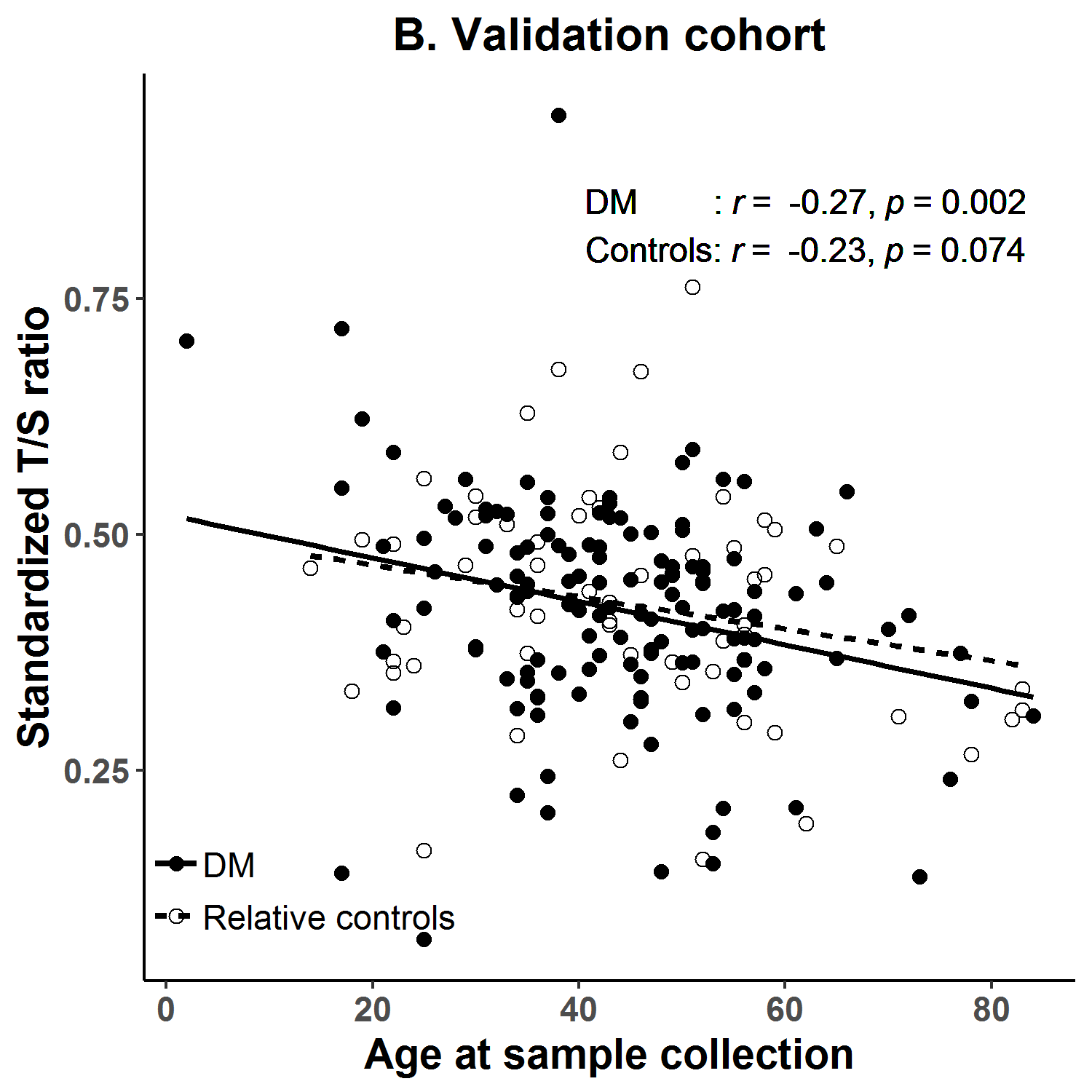** |
| --- | --- |

| **Supplemental Table 1. Baseline characteristics of DM1 patients and their unaffected relative controls, stratified by DNA extraction method.** | | | | |
| --- | --- | --- | --- | --- |
|  | Discovery cohort | | Validation cohort | |
| Characteristics | DM  (n=223) | Relative controls  (n=162) | DM  (n=138) | Relative controls  (n=61) |
| Age, median (range) | 40 (0-89) | 38.5 (6-89) | 43.5 (2-84) | 44 (14-83) |
| CTG repeat size, median (range)^1^ | 433  (43-2333) |  | 500  (50-1667) |  |
| Muscular Impairment Rating Scale^2^ |  |  |  |  |
| 1 | 38 (33%) |  | 25 (19%) |  |
| 2 | 25 (22%) |  | 26 (20%) |  |
| 3 | 30 (26%) |  | 46 (35%) |  |
| 4 | 22 (19%) |  | 32 (24%) |  |
| 5 | 1 (1%) |  | 2 (2%) |  |
| Male, n (%) | 98 (44%) | 67 (41%) | 67 (49%) | 31 (51%) |
| Calendar year at sample collection, n (%) |  |  |  |  |
| 1989-2001 | 60 (27%) | 54 (33%) | 4 (3%) | 0 (0%) |
| 2002-2005 | 82 (37%) | 48 (30%) | 0 (0%) | 0 (0%) |
| 2006-2011 | 65 (29%) | 53 (33%) | 15 (11%) | 13 (21%) |
| 2012-2016 | 16 (7%) | 7 (4%) | 119 (86%) | 48 (79%) |
| 1. CTG repeat size are unknown in 43 DM1 patients (discovery cohort n=26, validation cohort n=17). 2. Muscular Impairment Rating Scale (MIRS) was unknow or not clearly defined in 114 DM1 patients (discovery cohort n=107, validation cohort n=7). | | | | |

| **Supplemental Table 2. Comparison of baseline characteristics of DM1 patients with and without repeated relative telomere length measurement.** | | | | | |
| --- | --- | --- | --- | --- | --- |
|  | DM1 patients | | | |  |
| Characteristics | No  subsequent sample (N=349) | | With  subsequent sample (N=12) | | p |
| Age, median (min, max) | 42.0 | (0, 89.0) | 37.5 | (22.0, 59.0) | 0.50 ^1^ |
| CTG repeat size^2^, median (range) | 500.0 | (43.0, 2333.0) | 167.0 | (45.0, 1333.0) | 0.17 ^1^ |
| Muscular Impairment Rating Scale^3^ |  |  |  |  | 0.28 ^4^ |
| 1 | 58 | (25%) | 5 | (42%) |  |
| 2 | 48 | (20%) | 3 | (25%) |  |
| 3 | 72 | (31%) | 4 | (33%) |  |
| 4 | 54 | (23%) | 0 | (0%) |  |
| 5 | 3 | (1%) | 0 | (0%) |  |
| Male, n (%) | 158 | (45%) | 7 | (58%) | 0.37 ^5^ |
| Calendar year at sample collection, n (%) |  |  |  |  | 0.01 ^4^ |
| 1989-2001 | 60 | (17%) | 4 | (33%) |  |
| 2002-2005 | 78 | (22%) | 4 | (33%) |  |
| 2006-2011 | 76 | (22%) | 4 | (33%) |  |
| 2012-2016 | 135 | (39%) | 0 | (0%) |  |
| 1. Wilcoxon rank-sum test 2. CTG repeat size are unknown in 43 DM patients (discovery cohort N=26, validation cohort N=17). 3. Muscular Impairment Rating Scale (MIRS) was unknow or not clearly defined in 114 DM1 patients (discovery cohort n=107, validation cohort n=7). 4. Fisher’s exact test 5. Chi-square test | | | | | |

| **Supplemental Table 3. Telomere length attrition in DM1 patients and controls** | | | | | | |
| --- | --- | --- | --- | --- | --- | --- |
|  | Discovery cohort  (DM1 N=7, controls N=162) | | | Validation cohort  (DM1 N=5, controls N=61) | | |
| Effect | ß | SE | p | ß | SE | p |
| Intercept | 0.798 | 0.029 | <.0001 | 0.526 | 0.046 | <.0001 |
| DM1 *vs*. controls | 0.420 | 0.155 | 0.031 | 0.104 | 0.130 | 0.468 |
| Age | -0.005 | 0.001 | 0.0001 | -0.002 | 0.001 | 0.134 |
| DM*Age | -0.008 | 0.003 | 0.036 | -0.003 | 0.003 | 0.333 |
| Sex | -0.017 | 0.021 | 0.437 | -0.047 | 0.029 | 0.178 |
| ^*^Models included age, DM, age-DM interaction term and sex. | | | | | | |
